# Tumor Necrosis Factor-Alpha Induced by Hepatitis B Virus Core Mediating the Immune Response for Hepatitis B Viral Clearance in Mice Model

**DOI:** 10.1371/journal.pone.0103008

**Published:** 2014-07-21

**Authors:** Horng-Tay Tzeng, Hwei-Fang Tsai, I-Tsu Chyuan, Hsiu-Jung Liao, Chun-Jen Chen, Pei-Jer Chen, Ping-Ning Hsu

**Affiliations:** 1 Graduate Institute of Immunology, College of Medicine, National Taiwan University, Taipei, Taiwan; 2 Department of Internal Medicine, Taipei Medical University Shuang Ho Hospital, Taipei, Taiwan; 3 Institute of Clinical Medicine, College of Medicine, Taipei Medical University, Taipei, Taiwan; 4 Department of Internal Medicine, National Taiwan University Hospital, Taipei, Taiwan; 5 Department of Biochemical Science and Technology, National Taiwan University, Taipei, Taiwan; 6 Department of Microbiology, College of Medicine, National Taiwan University, Taipei, Taiwan; 7 Graduate Institute of Clinical Medicine, College of Medicine, National Taiwan University, Taipei, Taiwan; Drexel University College of Medicine, United States of America

## Abstract

Persistent hepatitis B viral (HBV) infection results in chronic hepatitis, liver cirrhosis, and hepatocellular carcinoma (HCC). An efficient control of virus infections requires the coordinated actions of both innate and adaptive immune responses. In order to define the role of innate immunity effectors against HBV, viral clearance was studied in a panel of immunodeficient mouse strains by the hydrodynamic injection approach. Our results demonstrate that HBV viral clearance is not changed in IFN-α/β receptor (IFNAR), RIG-I, MDA5, MYD88, NLRP3, ASC, and IL-1R knock-out mice, indicating that these innate immunity effectors are not required for HBV clearance. In contrast, HBV persists in the absence of tumor necrosis factor-alpha (TNF-α) or in mice treated with the soluble TNF receptor blocker, Etanercept. In these mice, there was an increase in PD-1-expressing CD8^+^ T-cells and an increase of serum HBV DNA, HBV core, and surface antigen expression as well as viral replication within the liver. Furthermore, the induction of TNF-α in clearing HBV is dependent on the HBV core, and TNF blockage eliminated HBV core-induced viral clearance effects. Finally, the intra-hepatic leukocytes (IHLs), but not the hepatocytes, are the cell source responsible for TNF-α production induced by HBcAg. These results provide evidences for TNF-α mediated innate immune mechanisms in HBV clearance and explain the mechanism of HBV reactivation during therapy with TNF blockage agents.

## Introduction

Hepatitis B virus (HBV) infection causes acute and chronic necroinflammatory hepatitis. In an acute HBV infection, cessation of virus replication and clearance of viral transcripts and antigens from the liver depends on HBV-specific cytotoxic T cells [1], [2]. An efficient control of virus infections requires the coordinated actions of both innate and adaptive immune responses [3]–[5], [6]. Innate immunity induces an antiviral state in infected cells by producing type I interferons (IFN), and supports the efficient maturation and site recruitment of adaptive immunity through the production of pro-inflammatory cytokines, in particular, tumor necrosis factor-α (TNF-α) [4]. However, in chronic HBV infection, impaired HBV-specific immune responses failed to eliminate infected hepatocytes, resulting in the persistence of HBV.

Experimental viral infection in both chimpanzees and woodchucks found only limited or even non-activation of innate immunity being demonstrated in acute HBV infection [3], [5], [7], [8]. Nevertheless, a transient though slight activation of IFN-α genes was detected in human hepatocytes infected by HBV in chimeric mice [9], in support of the innate immunity to sense and react to HBV. However, the mechanisms responsible for sensing HBV within the infected cells have not been elucidated yet, and which molecular components of the HBV actually recognized by the pattern recognition receptors (PRR) triggering the antiviral response is still undefined. In addition, a number of recent studies [10]–[12] has been investigated in suggesting the involvement of NK cells in chronic HBV infection and they could play a role in liver damage during reactivation. The role of innate immunity in viral clearance during HBV infection is still not clear.

TNF plays has long been considered as a key cytokine in HBV eradication. Higher intrahepatic levels of TNF-α have been associated with the increased expression of HLA class I molecules and an enhanced CD8^+^ T cell response to HBV, which leads to the more effective destruction of HBV-infected hepatocytes [13]. In chronic HBV infection, CD8^+^ T cells lack the ability to secrete enough TNF to kill HBV-infected hepatocytes, the so-called “exhausted phenotype.” This is a functional HBV-specific CD8 T cell impairment that is detectable at the peak of the disease when the majority of HBV-specific CD8^+^ T cells are activated but have little ability to proliferate and are functionally exhausted, probably due to upregulation of programmed death (PD)-1 [14], [15]. Studies have demonstrated that genetic polymorphisms leading to lower constitutive or inducible TNF-α expression are related to an increased risk of progression toward chronic HBV infection [16], [17]. Clinically, an anti-TNF regimen also reportedly increased the number of cases of HBV reactivation [18], [19]. However, pro-inflammatory cytokines are often undetectable during the early phases of HBV infection, and when present their production is lower during HBV infection [7], [20].

In this study, we investigated the role of TNF in viral clearance and persistence in a murine model of HBV persistence [21]–[23]. The model was used to identify the viral antigen crucial for HBV persistence, and it demonstrated that knocking out HBcAg led to HBV persistence in mice [22], indicating that HBcAg is critical in determining HBV persistence [22]–[24]. We demonstrate here that a deficiency of TNF-α reduces viral clearance and increases HBV persistence in this mouse model, indicating that TNF is crucial for mounting an effective anti-HBV immune response.

## Materials and Methods

### Ethics Statement

All animal work have been conducted according to relevant national and international guidelines. Mice were housed in pathogen-free facilities. They were housed in plastic cages at 25°C. All animals were monitored every 6–8 hours for signs of distress and endpoints including hunching, decreased socialization, anorexia, weight loss of 15% or more, the inability to evade handling. All hydrodynamic injections were performed under anesthesia, and all efforts were made to minimize suffering. For sacrifice, animals were humanely euthanized via CO2 asphyxiation followed by cervical dislocation and were considered non-survivors.

### Animal

BALB/c and C57BL/6 mice were obtained from National Laboratory Animal Center, Taiwan. Interferon-α receptor (IFN-αR) knockout mice were kindly provided by Dr. Guann-Yi Yu (National Health Research Institute, Taiwan). TNF-α knockout mice were obtained from Dr. Nien-Jung Chen (National Yang-Ming University, Taiwan). The ASC and NALP-3 knockout mice were obtained from Dr. Jenny Ting (University of North Carolina, NC). The RIG-I and MDA-5 knockout mice were obtained from Dr. Akira (Osaka University, Japan). The IL-1 receptor (IL-1R) knockout nice were obtained from Jackson laboratory (Bar Harbor, ME). All mice were maintained under SPF condition. Mice at 6–8 weeks of age were used. All animal experiments were performed according to the regulations approved by the Animal Ethical Committee of National Taiwan University.

### Hydrodynamic injection (HDI)

C57BL/6 mice and BALB/c (male, 6∼7 weeks old) were anesthetized with ketamine and xylazine. Ten micrograms of HBV plasmid DNA in a volume equivalent to 8% of the mouse body weight was injected via a tail vein in 5**s. Plasmid DNA was purified by using EndoFree Maxi plasmid kit (Qiagen, Hilden, Germany). For the trans-complementation assay, 10**µg of pAAV/core-null was combined with 10**µg of pFLAG-CMV2/HBc or pFLAG-CMV2/HBcY132A mutant [25]. Serum HBsAg were assayed as indicated time points to monitor the state of HBV persistence.

### Preparation of intrahepatic leukocytes

Livers were perfused with 0.2% bovine serum albumin (BSA)/phosphate-buffered saline (PBS), passed through a nylon mesh, and digested in collagense-IV and DNase-I (Sigma-Aldrich, St. Louis, MO) for 30**min. Hepatocytes were removed by centrifugation for 5**min at 100**g and washed with 0.2% BSA/PBS twice at 100**g for 5**min. The supernatant containing intrahepatic leukocytes (IHLs) was pelleted by 300**g centrifugation at 4°C for 10**min. Cell pellets were resuspended in 40% HBSS and layered in upper 70% percoll (GE Healthcare, Piscataway, NJ) gently. Next, cells were centrifuged at 1200**g for 20**min at 25°C. IHLs were collected, washed with 15**mL HBSS, and centrifuged at 300**g for 10**minutes at 4°C. Cell pellets were collected for further applications.

### Detection of the HBV antigen, antibody (Ab), and DNA

Serum levels of HBsAg, HBeAg, anti-HBc, and anti-HBs Abs were determined using the AXSYM system kit (Abbott Diagnostika, Wiesbaden, Germany). The cutoff value for determining HBsAg positivity was a signal-to-noise (S/N) ratio of ≥2 and a signal-to-cutoff (S/CO) ratio of ≥1. To detect serum HBV DNA, each serum sample was pretreated with 25 units of DNase I (Roche Diagnostics, Mannheim, Germany) at 37°C overnight, and total DNA was extracted and HBV DNA was detected by real-time PCR [21], [22]. Serum alanine transferase (ALT) was measured on a TBA-200FR automated clinical chemistry analyzer (Toshiba, Tokyo, Japan) [21], [22].

### Flow cytometry

For flow cytometry, allophycocyanin (APC)-conjugated anti-mouse CD3 (BD Biosciences, Palo Alto, CA), phycoerythrin (PE)-conjugated anti-mouse PD-1, fluorescein isothiocyanate (FITC)-conjugated CD127, and PE-Cy5.5-conjugated anti-mouse CD4 or CD8 (BD Biosciences Pharmingen) monoclonal (m) Abs were used for flow cytometry. For the flow cytometric analysis, 10^5^ cells were labeled in a fluorescence-activated cell sorter (FACS) buffer (PBS/2% FCS/0.1% sodium azide), fixed in 1% paraformaldehyde (Sigma*-*Aldrich, St. Louis, MO), and analyzed on a FACSCalibur using CellQuest software (Becton Dickinson, Mountain View, CA).

### Liver tissue preparation and immunoblotting

Mice were anesthetized and then subjected to intracardiac perfusion with PBS prior to liver tissue collection. For Southern blotting, 50**mg of mouse liver tissue was lysed in 700**µl of DNA extraction buffer (10**mM Tris-HCl, pH**8.0, 100**mM NaCl, 1**mM EDTA, 0.5% SDS, 0.5**mg of proteinase K) at 37°C overnight. Mouse DNA was digested with 40 units of HindIII (NEB) at 37°C overnight before gel electrophoresis. RNA was extracted by TRIzol (Invitrogen, Carlsbad, CA) according to manufacturer instructions. Purified 10**µg RNA was used for Northern blotting. Both Southern and Northern blot analyses were performed using a digoxigenin (DIG)-labeled probe system. DIG-HBx and DIG–mouse glyceraldehyde-3-phosphate dehydrogenase (DIG-mGAPDH) probes were used to analyze HBV DNA/RNA and mouse GAPDH mRNA, respectively.

For Western blotting of HBcAg, fifty micrograms of protein lysates were separated by 15% SDS-PAGE and then transferred to a PVDF membrane. The membrane was blotted by using rabbit anti-HBcAg (LTK BioLaboratories), mouse anti-β-actin (Sigma-Aldrich, St. Louis, MO), and horseradish peroxidase (HRP)-conjugated goat anti-rabbit (Promega, Madison, WI). The membrane was developed in an ECL system (Amersham Biosciences, Arlington Heights, IL).

### Immunohistochemistry

The perfused liver samples were embedded in optimal cutting temperature compound (OCT). Intrahepatic HBcAg or HBsAg were detected by immunohistochemical staining with rabbit anti-HBc (Dako, Glostrup, Denmark) or anti-HBs antibodies (Biomeda, Foster City, CA) and Envision System, HRP (DAB) (Dako, Glostrup, Denmark). Hematoxylin was used to stain liver section nuclei.

### Interferon (IFN)-γ enzyme-linked immunospot (ELISpot) assay

At the indicated time points after hydrodynamic injection, liver mononuclear cells were cultured and assayed for the frequency of antigen-specific IFN-γ- secreting cells using an ELISPOT kit (BD Biosciences, San Jose, CA). Briefly, 10^5^ liver mononuclear cells were co-cultured with 1**µg/ml of rHBcAg (ID Labs, London, Canada) in 200**µl RPMI 1640 supplemented with 10% fetal calf serum (FCS). Cell suspensions were incubated for 20**h. Spot-forming cells were revealed with a biotin-conjugated antibody, streptavidin-horseradish peroxidase (HRP), and AEC substrates (Sigma*-*Aldrich), and were analyzed using the ImmunoSpot series 5 analyzer (Cellular Technology Limited, Cleveland, OH).

### Preparation of recombinant FLAG-tagged HBc protein

Human 293 cells were transfected with FLAG-tagged WT HBc or HBcY132A-expressing vectors for 48 hours by Effectene according to the manufacturer’s instruction (Qiagen, Venlo, Netherlands). Cell lysates were harvested and subjected into immuno-precipitation by anti-FLAG sepharose (Sigma-Aldrich, St. Louis, MO) at 4°C with gentle rotation for 2 hours. After extensive washing by lysis buffer, the FLAG-HBc or FLAG-Y132A were eluted by 3xFLAG peptide (Sigma-Aldrich, St. Louis, MO) for 30**min.

### Co-culture assay

C57BL/6 mice were injected with pAAV/HBV1.2 or pAAV/core-null plasmids hydrodynamiclly. The hepatocytes were isolated at 3 days postinjection. The intrahepatic leukocytes were prepared from naïve C57BL/6 mice and cocultured with hepatocytes for 24 hours. Cell-culture supernatants were collected and TNF-α levels were detected by ELISA kit according to manufacturer’s instructions (eBioscience, San Diego, CA).

## Results

### The TNF-α rather than IFN-mediated pathway is critical in HBV clearance

**Figure 1:**
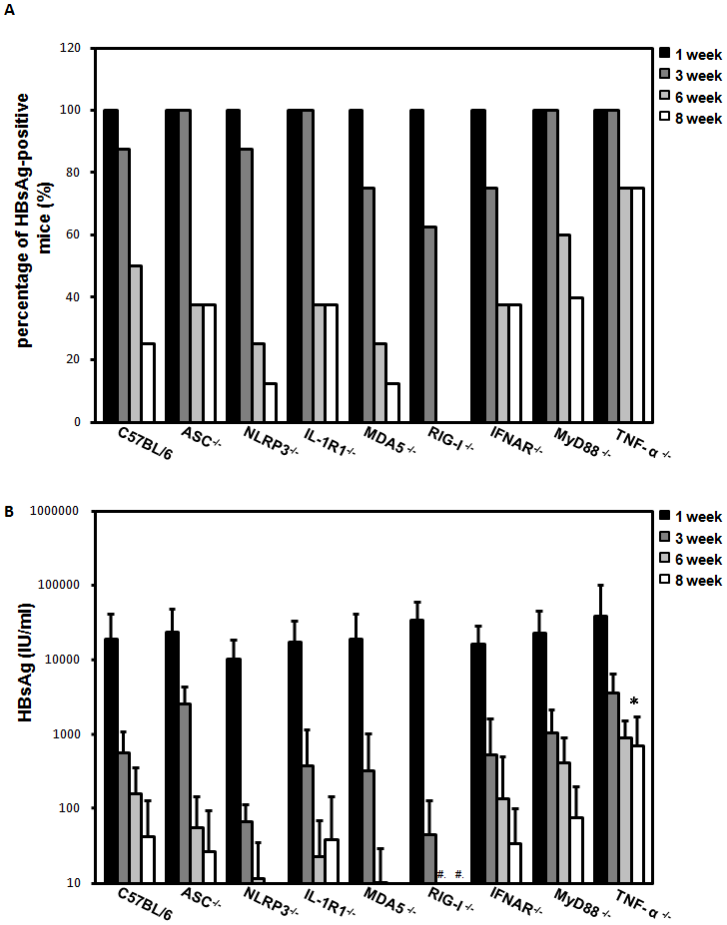
Prolonged HBV persistence in TNF-α knockout mice. Wild type C57BL/6 and different knockout mice, including NLRP3, apoptosis- ASC, MYD88, IL-1R, IFNAR, RIG-1, MAD5, and TNF-α, were introduced to the pAAV/HBV1.2 plasmid by hydrodynamic injection. HBsAg levels in mice serum were determined by an ELISA. HBsAg-positivity was defined as an S/N ratio greater than 2. The percentage of HBsAg-positivity (A) and serum HBsAg levels (B) were measured at Weeks 1, 3, 6 and 8. #, not detectable.

**Figure 2:**
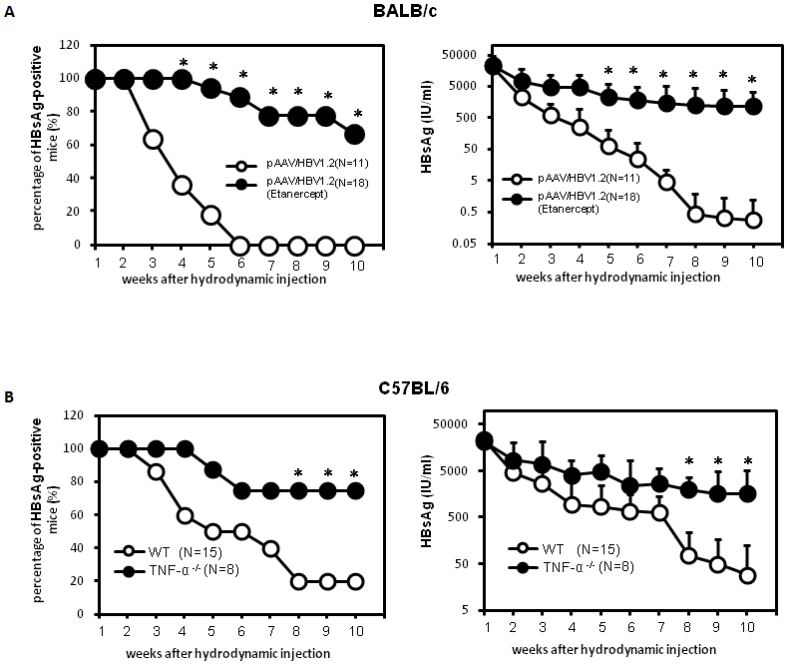
Delayed HBsAg clearance in mice with TNF deficiency. (A) BALB/c mice were treated with the recombinant soluble TNF-α receptor, Etanercept, on the day before hydrodynamic injection of the pAAV/HBV1.2 plasmid. Etanercept treatment was performed twice a week over the detection period. HBsAg levels in mice serum were determined by an ELISA. HBsAg-positivity was defined as an S/N ratio greater than 2. Differences in percentages (left) and serum levels (right) of HBsAg-positive mice with or without Etanercept were quantified. (B) Wild type C57BL/6 or TNF-α knockout mice were hydrodynamically transfected with the pAAV/HBV1.2 plasmid. Differences in percentages (left) and serum levels (right) of HBsAg-positive mice were quantified. *p<0.05.

Although the chronicity of HBV infection is the result of impaired HBV-specific immune responses that cannot efficiently eliminate or cure infected hepatocytes, however, this likely a result from the failure of immune responses at the first exposure to HBV. Therefore, we tested a panel of KO mice with specific deficiency in the innate immune sensors or effectors for their capability in clearing HBV after hydrodynamic injection (HDI) of a replication competent HBV DNA. This approach generated HBV persistence in C57BL/6 mice but not in BALB/c mice [21]–[23]. To identify the immune effectors of innate immunity that eliminate HBV from the liver, we monitored the persistence of HBsAg in a panel of gene knockout mice, including Nod-like receptor family protein 3 (NLRP3), apoptosis-associated speck-like protein containing a caspase recruitment domain (ASC), myeloid differentiation primary response gene 88 (MYD88), IL-1 receptor (IL-1R), IFN-α/β receptor (IFNAR), and TNF-α. The results (Figure 1) demonstrate that HBV viral clearance is not significantly different from wild type C57BL/6 mice in IFNAR, RIG-I, MDA5, MYD88, NLRP3, ASC, and IL-1R knock-out mice, indicating that these effectors are not required for HBV clearance. In contrast, only TNF-α knockout mice showed a markedly higher HBV-positive rate and prolonged HBV persistence compared to other strains, suggesting that TNF-α is an important effector cytokine that is required to clear HBV from the liver. The results (Figure 1 and Figure S1) demonstrated that the HBV persistence rate and serum HBs Ag levels was similar between IFNR knock-out mice and wild type C56BL/6 mice, indicating that IFN-mediated pathways are not essential for clearing HBV in this animal model. We further investigated the roles of innate cytokines TNF-α during HBV infection (Figure 2). There were very little or no TNF-α production could be detected in the liver in mice receiving pAAV/HBV1.2 plasmid (Figure S2). However, in contrast to IFNR knock-out mice, significantly impaired HBV clearance and enhanced HBV persistence was observed when TNF-α was neutralized with the soluble TNF receptor Etanercept in HBV-cleared mouse strain BALB/c, suggesting that TNF-α is required for HBV clearance (Figure 2A). Similarly, the HBV persistence rate and serum HBs Ag levels was significantly enhanced in TNF-α knockout mice compared to wild-type C57BL/6 mice (Figure 2B). Taken together, these results indicate that TNF rather than the IFN-mediated pathway is required for HBV clearance, and TNF blockage enhances HBV persistence *in vivo*.

### TNF-α deficiency leads to cytotoxic T lymphocyte dysfunction against HBV

**Figure 3:**
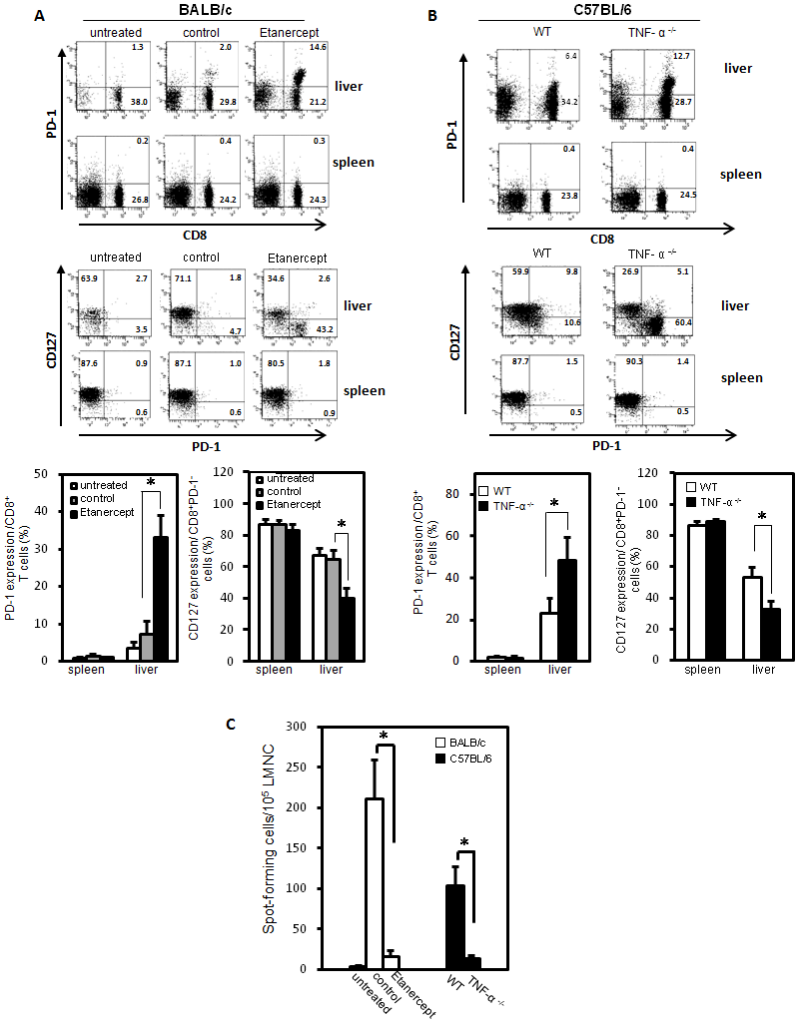
Liver-infiltrating CD8^+^ lymphocytes in Etanercept-treated and TNF-α knockout mice displayed the PD-1^hi^CD127^low^-exhausted phenotype and impaired HBcAg-specific IFN-γ T cell response. (A) BALB/c mice were hydrodynamically injected with WT pAAV/HBV1.2 plasmids in the presence or absence of Etanercept treatment. Eight weeks after injection, intrahepatic lymphocytes from HBsAg-positive mice were isolated and the PD-1, CD127 expressions of liver-infiltrating CD8^+^ lymphocytes and splenocytes were analyzed by flow cytometry. (B) Wild type C57BL/6 and TNF-α knockout mice were hydrodynamically injected with WT pAAV/HBV1.2 plasmids. Eight weeks after the injection, intrahepatic lymphocytes from HBsAg-positive mice were isolated and the PD-1, CD127 expressions by liver-infiltrating CD8^+^ lymphocytes and splenocytes were analyzed by flow cytometry. (C) Wild type C57BL/6 and TNF-α knockout mice as well as BALB/c mice with or without Etanercept treatment were hydrodynamically injected with pAAV/HBV1.2. Fourteen days after the injection, liver mononuclear cells (LMNCs) were isolated and HBcAg-specific IFN-γ responses were analyzed by an ELISpot assay. The frequency of HBcAg-specific IFN-γ-secretion was measured as spot-forming cells per 10^5^ LMNCs. *p<0.05. The data are representative of at least six independent experiments.

We then asked whether TNF-α deficiency is associated with an impaired T cell response to HBV in mice with HBV persistence. Recent studies in viral infection indicate that the interaction between the PD-1 on lymphocytes and its ligands plays a critical role in T-cell exhaustion by inducing T-cell inactivation and displayed lower levels of interleukin (IL)-7 receptor CD127, which had previously been described in association with the exhausted phenotype [26], [27]. The results in Figure 3A demonstrate that PD-1 is more highly expressed by intrahepatic CD8 cells in BALB/c mice hydrodynamically injected with HBV constructs and treated with Etanercept. Also, among intrahepatic PD-1 -expressing CD8 populations, CD127 expression was significantly reduced in mice treated with Etanercept. Interestingly, there are no such differences noted for the spleen. Our results indicate that there are significantly increased liver-infiltrating PD-1^hi^CD127^low^-exhausted CD8^+^ lymphocytes in Etancercept-treated HBV infected mice. Similar results were observed in TNF-α knockout mice (Figure 3B). Both results indicate that liver-infiltrating CD8^+^ lymphocytes in response to HBV in mice with TNF-α deficiency displayed the PD-1^hi^CD127^low^-exhausted phenotype.

We then evaluated T cell response to HBV in mice with HBV persistence after an infection. The HBV core-specific IFN-γ T-cell response in mice hydrodynamically injected with HBV DNA in the presence and absence of TNF-α were analyzed by an ELISpot assay. Results in Figure 3C demonstrate that the frequency of HBcAg-specific IFN-γ-secreting cells was significantly reduced in TNF-α knockout C57BL/6 mice or Etanercept-treated BALB/c mice. Taken together, our results indicate TNF-α is correlated with the anti-HBV T cell response *in vivo*.

### TNF blockage-induced elevation of serum HBV viral loads and maintained HBV viral gene expression within the liver

**Figure 4:**
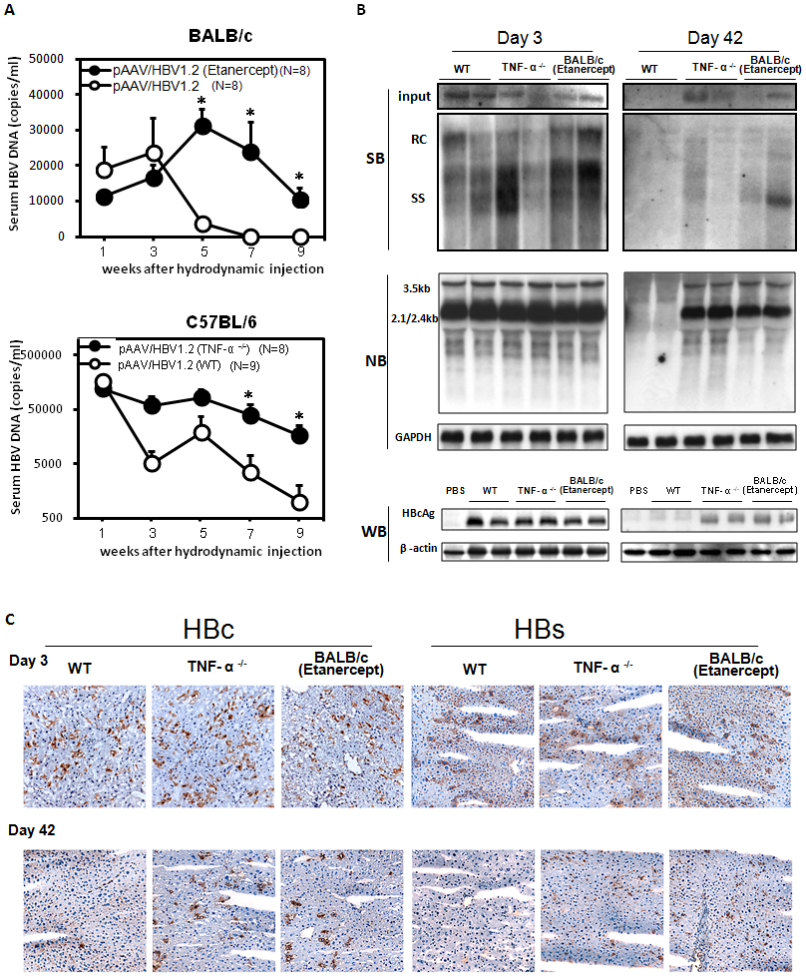
Delayed clearance of serum HBV DNA and increased viral gene expression in mice with TNF deficiency. (A) BALB/c mice with or without Etanercept treatment (upper panel) and TNF-α knockout mice (lower panel) were hydrodynamically injected with pAAV/HBV1.2. The serum HBV DNA in mice was quantified by real-time PCR at the indicated time points. The detection limit for HBV DNA in our system was 1000 copies per milliliter. (B) Liver samples were collected from Etanercept-treated BLAB/c and TNF-α knockout mice on Days 3 and 42 after pAAV/HBV1.2 hydrodynamic injection to examine viral replication, transcription, and HBcAg expression. Intrahepatic HBV DNA and viral transcripts were detected by Southern and Northern blotting, respectively, and GAPDH mRNA was shown as a loading control. The expressions of HBcAg and β-actin (loading control) were analyzed by SDS-PAGE followed by Western blotting. (C) Immunohistochemical staining for expressing HBcAg and HBsAg in the livers of Etanercept-treated BALB/c mice and TNF-α knockout mice compared to C57BL/6 (WT) mice on Days 3 and 42 after pAAV/HBV1.2 injection. *p<0.05.

Our results indicate that TNF-α deficiency is associated with the impaired T-cell response to HBV. We then investigated the effects of TNF blockage on HBV viral load and viral replication. The results (Figure 4A) show the control group BALB/c mice were almost cleared of HBV viral loads in the initial five weeks after hydrodynamic transfection of HBV. In contrast, persistently elevated HBV viral loads in serum were observed in BALB/c mice treated with Etanercept. TNF-α knockout mice showed similar results with persistently elevated HBV viral loads in serum compared to the wild type C57BL/6 mice. We then analyzed the HBV transcripts in the livers of mice transfected with HBV by Northern blotting after hydrodynamic injection (Figure 4B). The HBV transcripts remained detectable in the liver of TNF-α knockout and Etanercept-treated BALB/c mice up to Day 42 post-transfection. In contrast, the HBV transcripts were almost undetectable in wild type C57BL/6 mice on Day 42 post-transfection. Similarly, the results from an immunohistochemistry analysis also revealed that the staining for HBcAg and HBsAg remained detectable in the livers of TNF-α knockout and Etanercept-treated BALB/c mice on Day 42 post-transfection. However, HBcAg and HBsAg staining was much lower in wild type C57BL/6 mice on Day 42 post-transfection, which is correlated with serum viral loads and HBV transcripts in the liver (Figure 4C). Moreover, administration of TNF-α reduced serum HBsAg levels in mice hydrodynamically transfected with plasmid containing capsid assembly-defect mutant pAAV/Y132A or capsid deficiency pAAV/core-null mutant (Figure S3). These results indicate that TNF-α is responsible for HBsAg clearance and TNF-α deficiency impairs viral clearance and increases HBV viral load and viral replication *in vivo*.

### Lack of TNF-α eliminates HBcAg-induced HBsAg clearance

**Figure 5:**
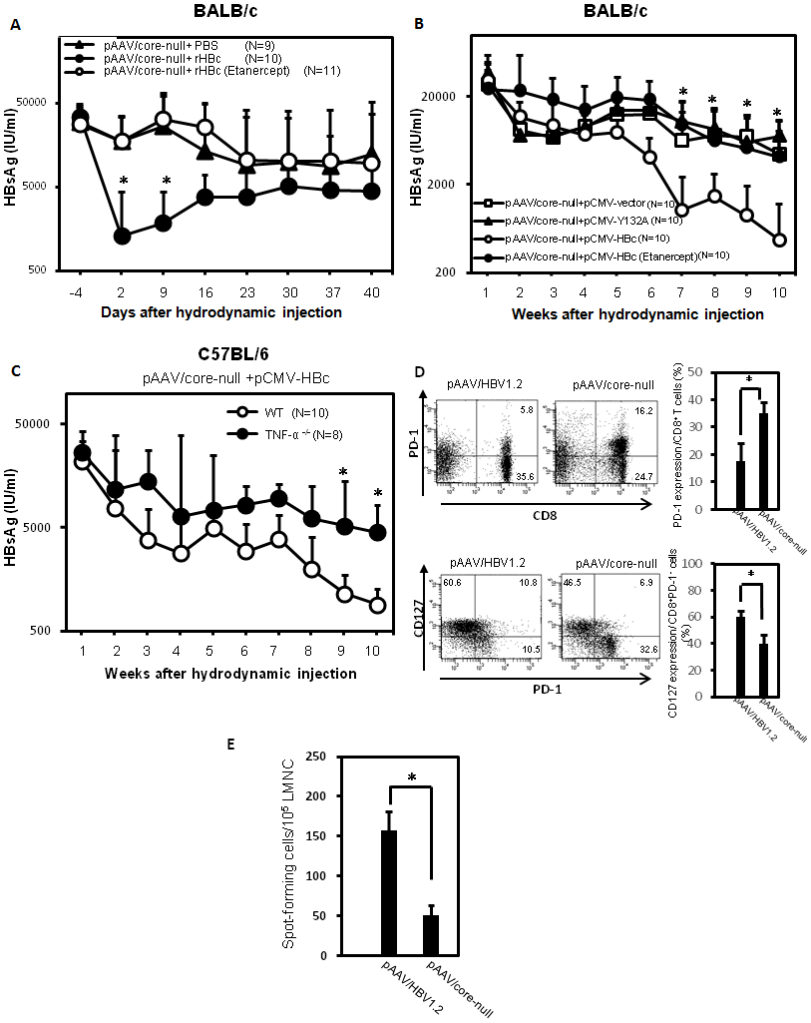
A lack of TNF-α abolished HBcAg-induced HBsAg clearance in mice sera. (A) BALB/c mice were hydrodynamiclly injected with pAAV/core-null plasmids, which contain a premature stop codon in the core open reading frame of a replication-competent HBV plasmid pAAV/HBV1.2 on Day 7. At Day 0, purified recombinant HBV core protein was injected hydrodynamically. Etanercept was administered on Days 1, 3, and 5 via intravenous injection. The serum HBsAg level was quantified at the indicated time points with an enzyme immunoassay [calculated as IU/ml]. N = number of mice in each experiment. (B) BALB/c mice were hydrodynamically injected with 10**µg of pAAV/core-null plus 10**µg of pFLAG-CMV2/HBc in the presence or absence of Etanercept or combined with pFLAG-CMV2/HBcY132A, a capsid assembly-defect mutant [25]. The titers of serum HBsAg were measured at the indicated time points. (C) C57BL/6 and TNF-α knockout mice were hydrodynamically injected with 10**µg pAAV/core-null plus 10**µg pFLAG-CMV2/HBc. The titers of serum HBsAg were measured [S/N]. (D) Intrahepatic lymphocytes from C57BL/6 mice hydrodynamically injected with pAAV/HBV1.2 or pAAV/core-null plasmid were isolated at 18 days postinjection. The expressions of CD8^+^ PD-1^+^ (upper panel) or levels of PD-1 or CD127 (lower panel) on CD8 T cells were analyzed by flow cytometry. (E) HBcAg-specific IFN-γ secretion of liver mononuclear cells from mice injected with pAAV/HBV1.2 or pAAV/core-null were analyzed by an ELISpot assay. The frequency of spot-forming cells were measured. Student’s-t test, *p<0.05.

Previous studies showed that the immune response triggered in mice by HBcAg during exposure to HBV is important in determining HBV clearance [22]–[24]. We then test whether HBcAg can induce TNF-α production *in vivo*. C57BL/6 mice were hydrodynamically injected with purified recombinant HBcAg. The results indicate the functional HBcAg/capsid could induce TNF-α production (Figure S4). To further define the role of TNF-α in the HBV core-induced HBV clearance in this mouse animal model, BALB/c mice were hydrodynamically injected with pAAV/core-null plasmid containing a core-deleted HBV construct [22] to induce persistent elevation of HBsAg in mice serum on Day-7, and then the mice were injected with purified recombinant HBV core protein on Day 0 in the presence or absence of Etanercept. Serum levels of HBsAg significantly decreased after injection with recombinant HBV core protein (Figure 5A). However, in mice treated with Etanercept, the effect of HBV core-induced clearance of HBsAg was eliminated. In addition, the introduction of a plasmid containing capsid assembly-defect mutant form of HBcY132A [25] failed to reduce the serum level of HBsAg (Figure 5B). However, co-injection of pAAV/core-null HBV plasmids with a plasmid encoding HBcAg efficiently decreased HBsAg levels. In contrast, when administered with Etanercept to neutralize TNF-α, the effect of HBV core-induced HBsAg clearance was abrogated. Similarly, the effect of HBV core-induced HBsAg clearance was also diminished in TNF-α knockout mice having elevated levels of HBsAg after hydrodynamic transfection with HBV (Figure 5C). To further confirm the T cell dysfunction in the pAAV/core-null transfected mice, the intrahepatic lymphocytes from mice receiving pAAV/HBV1.2 or pAAV/core-null were analyzed. Our results indicate there are significantly increased liver-infiltrating PD-1^hi^CD127^low^-exhausted CD8^+^ lymphocytes in mice infected with pAAV/core-null mutant (Figure 5D). Furthermore, the frequency of HBcAg-specific IFN-γ-secreting cells was significantly reduced in mice infected with pAAV/core-null mutant (Figure 5E). Taken together, these results indicate that TNF-α mediates the effects of HBV core-induced HBV clearance in this mouse animal model.

### Intra-hepatic leukocytes, in contrasting with HBcAg Containing hepatocytes, were responsible for HBcAg-induced TNF-α production

**Figure 6:**
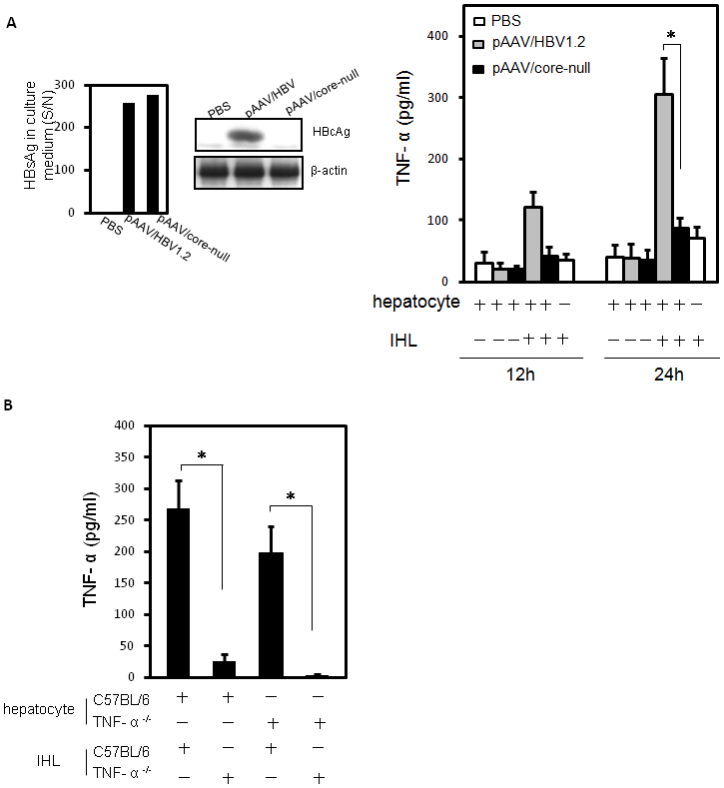
Intra hepatic leukocytes were responsible for HBcAg-induced TNF-α production in an *ex vivo* culture system. (A). C57BL/6 mice received pAAV/HBV1.2 or pAAV/core-null plasmids by hydrodynamic injection into a tail vein. At Day 3 post-injection, hepatocytes were isolated and cultured for 24**h. The secretory level of HBsAg and intracellular HBcAg expression were determined by enzyme immunoassay (upper left panel) and Western blotting (upper right panel), respectively. After 24**h of hepatocyte incubation, the intra hepatic leukocytes were isolated from naïve syngenic mice and co-cultured with hepatocytes for a further 12 or 24**h. The TNF-α production in culture medium was measured by ELISA assay. (B) Wild-type C57BL/6 or TNF-α knockout mice were injected hydrodynamically with pAAV/HBV1.2 plasmids. Hepatocytes were isolated and cultured for 24**h. Naïve intra hepatic leukocytes were isolated from wild-type C57BL/6 or TNF-α knockout mice and incubated with hepatocytes for 24**h. The TNF-α level in culture supernatants was determined by ELISA assay.

Our results indicate that TNF-α is required for an effective T-cell response to HBV and the immune response triggered in mice by HBcAg during exposure to HBV is critical in HBV clearance. We then investigated the cellular source of TNF-α, which is responsible for HBV core-induced HBV clearance. We used an *ex vivo* system with isolation of intra-hepatic leukocytes (IHL) from mice receiving pAAV/HBV1.2 or pAAV/core-null plasmids and co-cultured with primary syngeneic hepatocytes. The results (Figure 6) demonstrate that the IHLs, but not the hepatocytes, are the cell source responsible for TNF-α production induced by HBcAg in this mouse animal model. There was no TNF-α production when adding the IHLs isolated from mice receiving pAAV/core-null plasmids or from TNF-α deficient mice in this *ex vivo* co-culture. Taken together, our results indicate that the production of HBcAg-induced TNF-α by IHLs is required for an effective T cell response to HBV.

## Discussion

In this study, we demonstrate that a TNF blockade using a soluble TNF receptor, Etanercept, or mice with a TNF-α gene deficiency all suffer delayed viral clearance and enhanced HBV persistence in this mouse model. Previous studies revealed that several innate inflammatory cytokines had potential modulating effects on HBV gene expression, including IFN-α, IL-1α, IL-6 [28], and TNF-α [29]–[31]. However, due to experimental limitations, only limited or even non-activation of innate immunity could be demonstrated in acute HBV infection. The role of innate immunity on HBV clearance is still uncertain. To address the role of innate immune responses in HBV clearance, we examined a panel of knock-out mice by a single hydrodynanmic injection of HBV DNA. In this study, after examining the gene-specific KO mice panels, we found the three major innate pathways (RIG-I, NOD, inflammasome) to be dispensable for HBV clearance. However, a deficiency of TNF-α reduces viral clearance and increases HBV persistence in this mouse model, indicating that the innate cytokine TNF-α is crucial in HBV clearance. The role of the immune effectors required in HBV viral clearance has been studied previously in a panel of immunodeficient mouse strains [6]. However, only CD8^+^ T cells were determined to be the key cellular effectors mediating HBV clearance from the liver, and the roles of TNF and other innate effectors in HBV viral clearance were not addressed [6]. To clarify the effects of innate effectors on HBV clearance, we used a panel of innate effector knockout mice to study their effects on HBV persistence in this mouse model. Our results indicate that HBV triggers innate immunity via a TNF-dependent process to induce an effective T cell response to HBV. The results are consistent with findings in previous study by Yang et. al. concluding that TNF is required to eliminate both the HBV DNA and HBcAg from the liver [6]. Although IFN-α/β receptor deficiency mice seemed to be impaired in their ability to eliminate the transcriptional template from the liver in that study, their ability to clear HBcAg remained intact [6], suggesting that the IFN pathway was not sufficient to clear the HBV and therefore might play a supporting role in HBV clearance. This is consistent with the results of other studies in humans and animal models that type I IFNs are often undetectable during the early phases of HBV infection, and when present their production is decreased [7], [20]. Accumulating evidence suggests that HBV infection induces host immunotolerance [21], [32]. Persistent HBV infection sustains the suppression of antiviral immunity, and high HBV titers or particle loads can inhibit innate immune response activation, particularly innate PRRs and their downstream signals in hepatocytes [3], [33], [34]. Nevertheless, in support of the innate immunity to sense and react to HBV, in this study, our results indicate that HBV triggers innate immunity via a TNF-dependent process to induce an effective T cell response to HBV.

As an innate cytokine, the induction of TNF-α not only triggers an inflammatory response but also activates adaptive immunity against HBV and may regulate the balance of virus replication and clearance within the liver. In this study, mice with TNF-α deficiency demonstrated an increase in exhausted-phenotype of CD8^+^ T cells and impaired T cell response to HBV. In addition, TNF blockade significantly increased the serum HBV DNA, the expression of HBV core, and HBV viral replication within the liver, indicating that TNF-α is crucial for mounting an effective anti-HBV immune response. TNF has direct anti-viral effect on HBV [31], [35]. In addition, TNF alpha is the key mediator that drives local intrahepatic proliferation of T cells (iMATEs: ‘intrahepatic myeloid-cell aggregates for T cell population expansion’) [36], and the effect of TNF in HBV clearance in this mouse model could be due to such iMATEs. Therefore, the effects of HBV clearance by TNF in this mouse system may not exclusively due to the direct anti-HBV effect, and it is likely due to triggering the innate immunity by HBcAg/capsid to induce T cell response to HBV, and both cytopathic and noncytopathic mechanisms are involved [35], [37], [38].

Our results also demonstrate that the HBV core is critical for inducing TNF-α to clear HBV and for TNF blockage to eliminate HBV core-induced viral clearance effects in mice. It implies that the HBV core induces TNF-α through an innate immune sensor to trigger a host anti-HBV immune response that leads to viral clearance. However, the mechanisms responsible for sensing HBV within the infected cells, and which molecular components of the HBV DNA, RNA or viral proteins are actually recognized by the PRR triggering the antiviral response is still undefined. In this study, our results demonstrate that the HBV core is critical for inducing TNF-α and enhancing the clearance of HBV. In addition, TNF blockage abolished the HBV core-induced viral clearance effects in mice, suggesting that host innate immunity senses the HBV core through the innate immune sensor and induces TNF-α. Taken together, these results indicate that blockage of TNF-α inhibits the effects of HBV core-induced HBV clearance in this mouse animal model. In addition, the introduction of a capsid assembly-defect mutant form of HBcY132A [25] failed to trigger the HBV core-induced viral clearance effects, suggesting the assembled viral capsid is critical for sensing HBV within the infected cells through the innate immune sensor. The innate sensors that recognize and bind to the HBV core or capsid still require further investigation.

Anti-TNF is effective for the treatment of rheumatoid arthritis, seronegative spondyloarthropathy, and inflammatory bowel disease. However, there are limited reports on its effect on HBV persistence and reactivation in chronic HBV infection during anti-TNF therapy [18], [19]. We demonstrate in this study that TNF blockage reduces viral clearance, induces elevated serum HBV viral loads, enhances HBV viral gene expression, and increases HBV persistence in a mouse model. Therefore, treatment with TNF blockage agents may reduce the clearance of HBV and enhance HBV replication and viral persistence. In conclusion, TNF-α deficiency significantly increased serum HBV DNA and viral replication within the liver, indicating that TNF-α is crucial for mounting an effective anti-HBV immune response. Thus, our results provide evidence that therapy with TNF blockage agents may impair the T cell response and enhance viral replication during chronic HBV infection.

## Supporting Information

Figure S1 **No significant difference in HBsAg clearance between wildtype C57BL/6 and IFNAR knockout mice.** C57BL/6 or IFNAR knockout mice were injected with the pAAV/HBV1.2 plasmid hydrodynamically. The serum level of HBsAg was determined weekly via an enzyme immunoassay [calculated as IU/ml]. N equaled the number of mice in each experiment. The positive rate of HBsAg (left panel) and serum HBsAg titers (rifht panel) were shown.
(JPG)

Figure S2 **Hepatic TNF-α expression was not induced in mice receiving hydrodynamical injection with pAAV/HBV1.2 plasmid.** C57BL/6 mice were hydrodynamically injected with pAAV vector, pAAV/HBV1.2 or pAAV/core-null plasmid. The liver samples were collected at the indicated time points. Total RNA and protein were extracted, and the TNF-α level in mice liver was analyzed by quantitative RT-PCR (A) and ELISA kit (B).
(JPG)

Figure S3 **Administration of TNF-α suppressed serum levels of HBsAg in mice with HBV persistence.** C57BL/6 mice were *in vivo* transfected with plasmid pAAV/HBVY132A (A) or pAAV/core-null (B) by hydrodynamic injection. The mice received intraperitoneal injection of 200,000 U of recombinant TNF-α every other day. The serum samples were collected at the indicated time points, and the titers of HBsAg were measured by immunoassay [IU/ml].
(JPG)

Figure S4 **Hepatitis B viral core capsid triggered TNF-α production** ***in*** ** ***vivo.*** HEK293 cells were transfected with FLAG tagged HBcAg-expressing plasmid. The FLAG-HBc was purified from cell lysates by anti-FLAG affinity gel. The FLAG-HBc absorption was performed by anti-HBc antibody precipitation [rHBc(−)] and analyzed by SDS-PAGE analysis. To verify the formation of FLAG-HBc capsid, the purified FLAG-HBc was subjected into native agarose gel electrophoresis followed by immunoblotting with anti-FLAG antibody. C57BL/6 mice were divided into four groups and administrated with the indicated reagents, including PBS solution, 20**µg recombinant FLAG-HBc (A) or FLAG-HBcY132A(B), preparation after anti-HBc antibody absorption [rHBc(−)], or 5**µg poly I:C by hydrodynamic injection, respectively. Serum samples were collected at the indicated time points. The levels of TNF-α was measured by ELISA kits.
(JPG)
