# Supplementary figures and images for: Tumor Necrosis Factor-Alpha Induced by Hepatitis B Virus Core Mediating the Immune Response for Hepatitis B Viral Clearance in Mice Model
Source: PLoS One. 2014 Jul 21;9(7):e103008. doi: 10.1371/journal.pone.0103008 (PMC4105421; doi:10.1371/journal.pone.0103008)

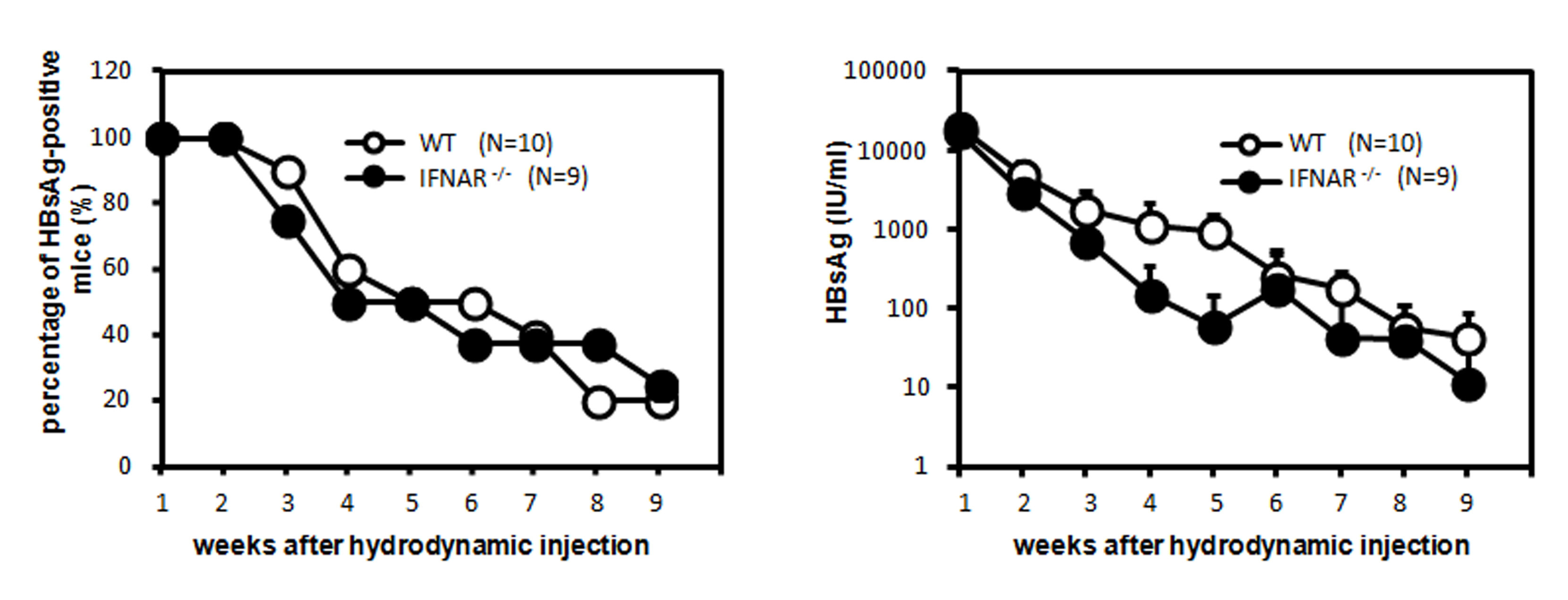

Supplement: Figure S1 — No significant difference in HBsAg clearance between wildtype C57BL/6 and IFNAR knockout mice. C57BL/6 or IFNAR knockout mice were injected with the pAAV/HBV1.2 plasmid hydrodynamically. The serum level of HBsAg was determined weekly via an enzyme immunoassay [calculated as IU/ml]. N equaled the number of mice in each experiment. The positive rate of HBsAg (left panel) and serum HBsAg titers (rifht panel) were shown. (JPG) [file pone.0103008.s001.jpg]

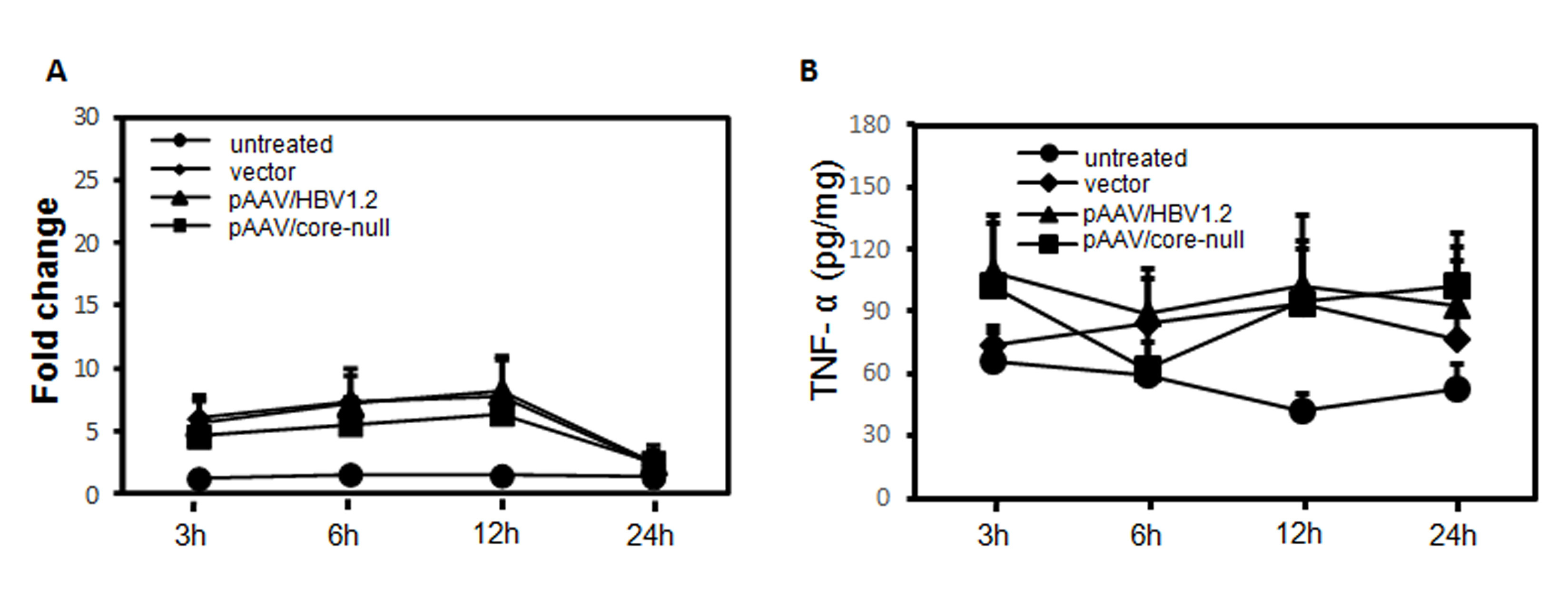

Supplement: Figure S2 — Hepatic TNF-α expression was not induced in mice receiving hydrodynamical injection with pAAV/HBV1.2 plasmid. C57BL/6 mice were hydrodynamically injected with pAAV vector, pAAV/HBV1.2 or pAAV/core-null plasmid. The liver samples were collected at the indicated time points. Total RNA and protein were extracted, and the TNF-α level in mice liver was analyzed by quantitative RT-PCR (A) and ELISA kit (B). (JPG) [file pone.0103008.s002.jpg]

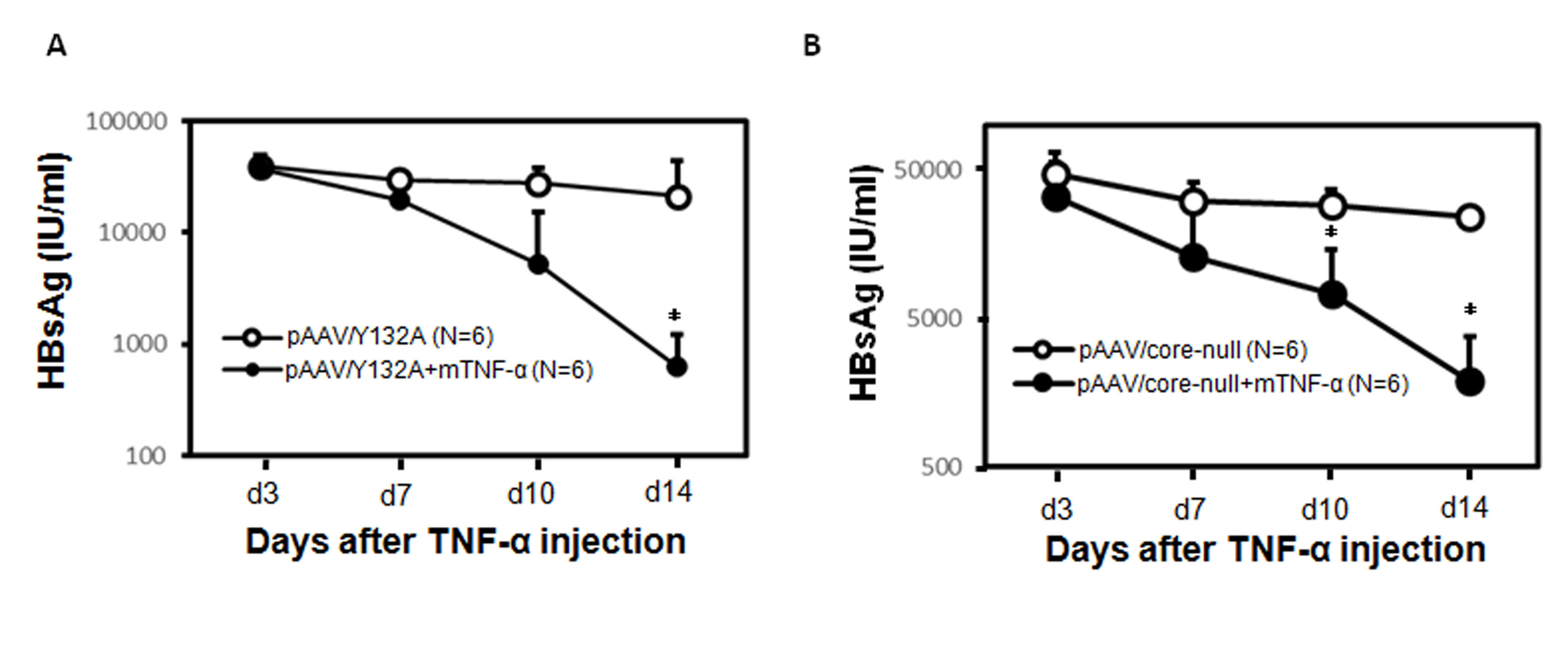

Supplement: Figure S3 — Administration of TNF-α suppressed serum levels of HBsAg in mice with HBV persistence. C57BL/6 mice were in vivo transfected with plasmid pAAV/HBVY132A (A) or pAAV/core-null (B) by hydrodynamic injection. The mice received intraperitoneal injection of 200,000 U of recombinant TNF-α every other day. The serum samples were collected at the indicated time points, and the titers of HBsAg were measured by immunoassay [IU/ml]. (JPG) [file pone.0103008.s003.jpg]

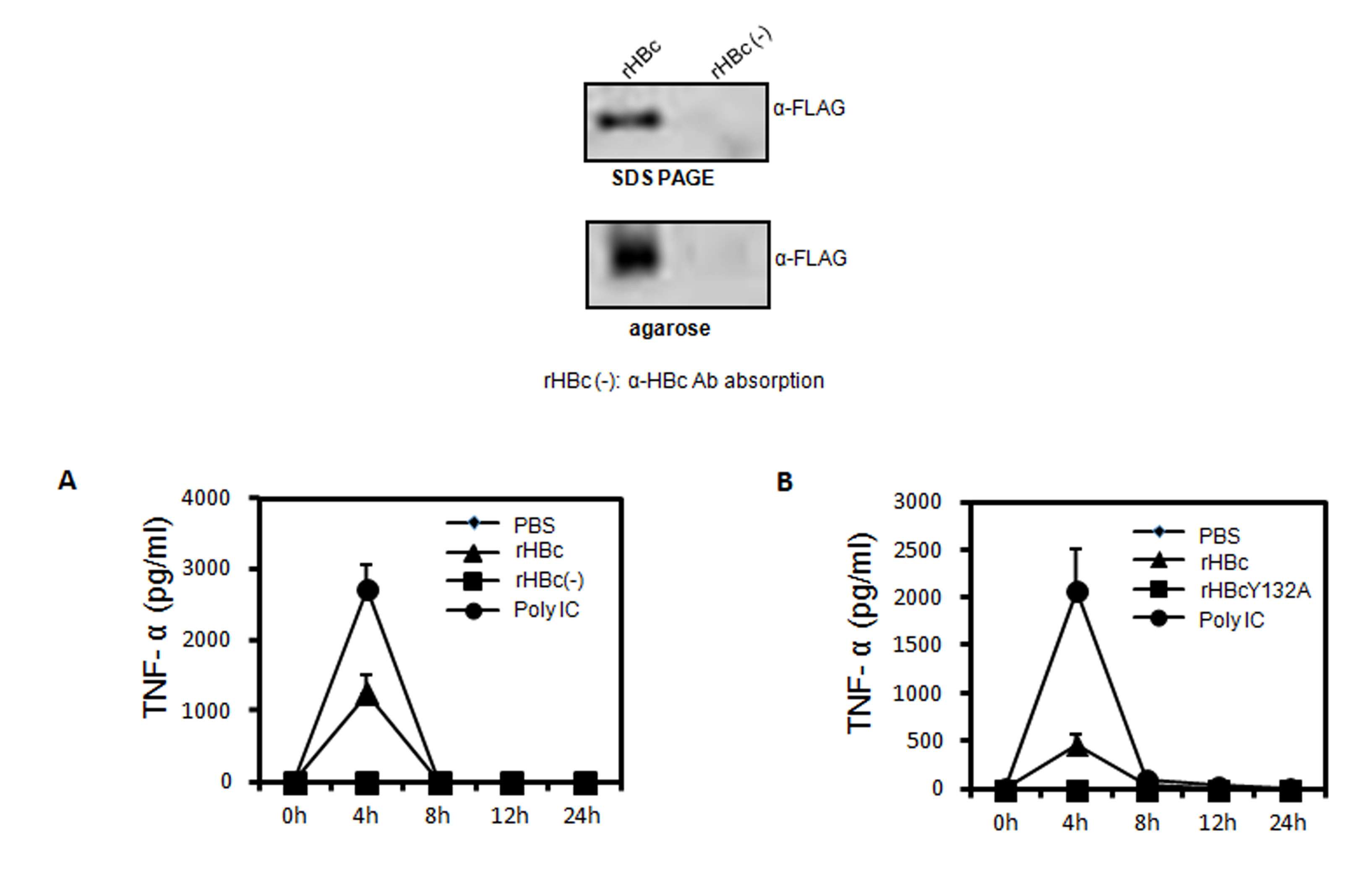

Supplement: Figure S4 — Hepatitis B viral core capsid triggered TNF-α production in vivo. HEK293 cells were transfected with FLAG tagged HBcAg-expressing plasmid. The FLAG-HBc was purified from cell lysates by anti-FLAG affinity gel. The FLAG-HBc absorption was performed by anti-HBc antibody precipitation [rHBc(−)] and analyzed by SDS-PAGE analysis. To verify the formation of FLAG-HBc capsid, the purified FLAG-HBc was subjected into native agarose gel electrophoresis followed by immunoblotting with anti-FLAG antibody. C57BL/6 mice were divided into four groups and administrated with the indicated reagents, including PBS solution, 20µg recombinant FLAG-HBc (A) or FLAG-HBcY132A(B), preparation after anti-HBc antibody absorption [rHBc(−)], or 5µg poly I:C by hydrodynamic injection, respectively. Serum samples were collected at the indicated time points. The levels of TNF-α was measured by ELISA kits. (JPG) [file pone.0103008.s004.jpg]
